# Supplementary material for: Maternal fibrinogen/fibrin degradation products to high density lipoprotein cholesterol ratio for predicting delivery of small and large for gestational age infants: a pilot study
Source: Lipids Health Dis. 2023 Dec 12;22:221. doi: 10.1186/s12944-023-01986-x (PMC10714553; doi:10.1186/s12944-023-01986-x)
Supplement: Supplementary file 1 — Supplementary Material 1 [file 12944_2023_1986_MOESM1_ESM.pdf]

Manuscript for iThenticate.docx

By IIT-A-231113497 IIT-A-231113497

WORD COUNT	3905	TIME SUBMITTED	17-NOV-2023 10:01AM
		PAPER ID	104313579

Maternal fibrinogen/fibrin degradation products to high density lipoprotein cholesterol ratio for predicting delivery of small and large for gestational age infants: A pilot study

Abstract

Background The purpose of this pilot study was to investigate associations between fibrinogen/fibrin degradation products (FDP) to high density lipoprotein-cholesterol (HDL-C) ratio (FHR) of mothers and the risk of delivering large/small for gestational age (LGA/SGA) infants and to evaluate the predictive power of FHR on LGA/SGA.

Methods This study retrospectively reviewed 11,657 consecutive women whose lipid profiles and FDP levels were investigated at the time of admission for delivery at a specialized hospital. The FHR was calculated, and perinatal outcomes, including clinical parameters, were analyzed.

Results The prevalence of SGA was 9% (n=1034), and that of LGA was 15% (n=1806) in this cohort study. FHR was significantly lower in women who delivered SGA infants (4.0 ± 3.2 vs. 4.7 ± 3.3 mg/mmol, $P < 0.01$) and higher in women who delivered LGA infants (5.7 ± 3.8 vs. 4.7 ± 3.3 mg/mmol, $P < 0.01$) compared with those who delivered infants of normal size for their gestational age. Women in the top quartile for FHR (>5.9 mg/mmol) had a 2.9-fold higher risk of delivering LGA infants [adjusted odds ratio (OR)=2.9, $P < 0.01$] and a 47% lower risk of delivering SGA infants (adjusted OR = 0.47, $P < 0.01$) than those in the bottom quartile (< 2.7 mg/mmol). In addition, adding FHR to the conventional models significantly improved the area under the curve for the prediction of delivering LGA (0.725 vs.

0.739, $P < 0.01$) and SGA (0.717 vs. 0.727, $P < 0.01$) infants.

Conclusion These findings suggest that the FHR calculated in late pregnancy is an innovative predictor of delivering LGA and SGA infants. Combining FHR with perinatal parameters could thus enhance the predictive ability for predicting the delivery of LGA/SGA infants.

Keywords FHR, FDP, HDL-C, birthweight, SGA, LGA, prediction

Introduction

Birth weight is often recognized as an index to evaluate foetal intrauterine growth. Abnormal foetal growth could affect the incidence of diseases in adulthood based on the theory that diseases that occur later on in life originate from when the foetus is in the womb [1]. Newborns who are large 2 and small for gestational age (LGA/SGA), generally referred to as having a birthweight above the 90th and below the 10th percentiles at particular gestational weeks, have an elevated risk of a variety of adverse outcomes during the perinatal period and from early life to adulthood [2]. LGA infants are prone to develop diabetes, future cancers and obesity, and have an early onset of cardiovascular diseases [3]. SGA infants are also known to show early signs of metabolic problems, abnormal body fat distribution, and a high risk of diabetes, heart disease, and neurodevelopmental disorders [4]. In recent years, an increased prevalence of SGA and LGA newborns has been reported in developing countries and developed countries, respectively [5,6]. Therefore, it remains important and challenging in modern antenatal care to prevent adverse pregnancy outcomes by detecting foetal growth disorders.

For foetuses to grow properly, there are a variety of maternal biological and physiological changes that must occur during pregnancy. Studies on indices of these modifications are becoming more popular. Until now, a number of factors, such as maternal micronutrients from one-carbon metabolism and glucolipid metabolites and coagulation-related parameters, have been correlated with neonatal birthweight [7–9]. However, the impacts of maternal biomarkers on LGA/SGA newborns during the uncomplicated pregnancies are inconsistent. For instance, some studies have found that maternal high density lipoprotein-cholesterol (HDL-C) during pregnancy is adversely associated with neonatal birthweight and LGA incidence [10–14], while other studies reported no significant associations [15,16]. On the other hand, fibrinogen/fibrin degradation products (FDPs) have traditionally been used to diagnose disseminated intravascular coagulation (DIC) [17]. To detect thrombophilia in pregnant women who suffered recurrent pregnancy loss with unknown causes, Wang et al. established a predictive model including FDP and other coagulation parameters [18]. A previous study reported that the plasma FDP level at admission for hospital delivery is associated with neonatal birthweight and LGA risk during normal pregnancy [19]. To date, few studies have reported a relationship between FDP level and SGA delivery.

The exploration of more efficient indicators appears to have important significance not only for foetal birthweight itself but also for the prediction of adverse LGA/SGA. It remains unclear whether a combination of FDP and HDL-C [FDP to HDL-C ratio (FHR)] can be a reliable indicator of LGA/SGA. The assumption of this

pilot research was that FHR is more efficient in predicting the delivery of LGA/SGA infants than using either FDP or HDL-C alone. To bridge this knowledge gap, the objective of this pilot study was to comprehensively assess the associations of maternal FHR, FDP, and HDL-C with foetal birthweight and the delivery of LGA/SGA infants among the Chinese population, and to evaluate the power of FHR before delivery to predict the delivery of LGA/SGA infants.

Methods

Study participants and data acquisition

From April 2016 to March 2017, an observational study was performed in Changzhou city, eastern China. A total of 13,275 consecutive pregnant women who delivered at Maternal and Child Health Care Hospital of Changzhou were initially included in this retrospective study to explore the associations between antenatal measurements of relative biomarkers and adverse birth outcomes. The inclusion criteria were as follows: 1) pregnancy at 28–41 gestational weeks; 2) singleton pregnancy and live birth; and 3) clearly completed medical records. The exclusion criteria were as follows: 1) use of illicit drugs and alcohol and smoker; 2) major prepregnancy diseases (chronic hypertension, diabetes mellitus, chronic liver, kidney and heart diseases, thyroid and immune rheumatic disease, and syphilis); 3) foetal congenital malformation; and 4) no results of FDP and HDL-C. A total of 1618 observational subjects were excluded from the present cohort for the following reasons: 1) 335 cases with multiple gestation; 2) 96 cases without live births; 3) 488 cases with pregestational diseases; and 4) 699 cases without FDP and HDL-C measurements (Fig.1). After the above exclusion

criteria, 11,657 subjects were included in the final analysis. Basic information on mothers and their newborns, including age, body mass index (BMI), parity, blood pressure (BP), medical history, bad habits, pregnancy complications, neonatal age, sex, weight, and height, was obtained from the hospital's medical records. Laboratory results were collected from the hospital's clinical chemistry database. All participants were admitted to the hospital for delivery when the onset of threatened labour occurred. Blood samples were taken after admission and transferred to the laboratory for routine measurements, including FDP, lipid profile, and high sensitivity C reaction protein (hsCRP). Plasma FDP levels were measured by an automatic automatic coagulometer with a matching reagent (Sekisui Medical, Japan and Thrombolyzer XRM, Behnk Elektronik, Germany). Serum lipids profile and hsCRP were detected by the automatic analysers with supporting reagents (lipid profile: Beckman Coulter, Japan; hsCRP: Siemens Diagnostics, Germany). Reference intervals for FDP, hsCRP, total cholesterol, triglyceride, low density lipoprotein-cholesterol (LDL-C) and HDL-C based on previous reports were 2.50–5.28, 0.60–19.16 mg/L, 5.73–7.44, 2.70–4.02, 1.74–2.32, and 2.95–4.31 mmol/L, respectively [20–22].

The study protocol was approved by the Ethics Committee in Maternal and Child Health Care Hospital of Changzhou (ZD201803). Anonymous data were analysed and written informed consent for observational subjects was waived in the present study.

Definitions

Maternal age was classified as advanced (≥35 years) or appropriate (<35 years) [23]. Pregnant women at admission were classified into obese (≥30.0 kg/m^2), overweight

(25–29 kg/m^2) and normal weight (<25 kg/m^2) according to the World Health Organization classification of BMI [12]. The diagnosis of pregnancy complications was described in a previous report and included gestational diabetes mellitus (GDM), intrahepatic cholestasis of pregnancy (ICP), pre-eclampsia (PE), pregnancy induced hypertension (PIH), and preterm birth [24]. According to the birthweight and gestational week, LGA and SGA were categorized as birthweights higher than the 90th and lower than the 10th percentiles of the gestational week-specific reference intervals [25].

Statistical analysis

The distributions of continuous variables were investigated by using the Kolmogorov–Smirnov test. The variables following skewed distributions were described as the median [inter-quartile range (IQR)], and those following normal distributions were expressed as the mean [standard deviation (SD)], and categorical variables were expressed as percentages. To compare the differences in anthropometric and laboratory parameters among the quartiles of maternal FHR, chi-square, Kruskale–Wallis and ANOVA tests were performed for categorical variables, nonnormally distributed continuous variables and normally distributed continuous variables, respectively. Spearman's test was applied to determine the correlation of FHR with other indices. The birth weight and length were first treated as continuous variables, and linear analysis models were applied to assess the impacts of different indices (FHR, FDP, and HDL-C) on these variables. When birth weight (LGA and SGA) was defined as a categorical variable, logistic analysis models were

applied to investigate associations of LGA/SGA with FHR, FDP, and HDL-C. Adjusted variables included age, BMI, blood pressure (BP), parity, gestational age, pregnancy complications, assisted reproduction, and foetal sex and the levels of total cholesterol, triglycerides, LDL-C and hsCRP. Similarly, odds ratios (ORs) [95% confidence interval (CI)] of FHR on LGA/SGA across each subgroup were calculated, and their interactions were determined. Receiver operating characteristic (ROC) curves were analysed to compare the area under the curve (AUC) calculated by FDP, HDL-C, FHR and models to predict the delivery of SGA and LGA infants. Youden's index was used to determine the cut-off value to predict the delivery of SGA and LGA infants.

Empower software (version 2.0, X&Y Solutions, USA) was used in all the statistical analyses. A two-tailed $P < 0.05$ indicated statistical significance.

Results

Population characteristics

The median age of the participants was 28 years old (range 15–47). Approximately 75% of observational subjects had a prenatal BMI ≥ 25 kg/m^2 and more than 60% of them were nulliparous. The median birth length and weight, and gestational week were 50 cm, 3337 g, and 39 weeks, respectively. Among the 11657 newborns, 1806 (15%) were classified as LGA, and 1034 (9%) were classified as SGA. Table 1 shows the demographic characteristics by quartiles of FHR. The ranges of FHR for quartiles (Q) 1–4 were <2.70, 2.70–3.98, 3.99–5.90, and >5.90 mg/mmol, respectively. Significant differences in terms of age, parity, BP, assisted reproduction, the prevalence of

pregnancy complications (PTB/GDM/ICP/PIH), birth length and weight, neonatal sex, and laboratory results (hsCRP/total cholesterol/triglyceride) were found among the quartiles, with the exception of BMI, the prevalence of PE, and LDL-C levels. A negative relationship between FHR quartiles and SGA prevalence was observed (13.0% in Q1, and decreased to 9.8%, 6.6%, and 6.1% in Q2, Q3, and Q4, respectively). Additionally, there was a positive relationship between FHR quartiles and LGA prevalence (9.4% in Q1, and increased to 14.1%, 16.5%, and 22.0% in Q2, Q3, and Q4, respectively). FDP, HDL-C, and the FDP:HDL-C ratio were significantly different among the pregnant women who delivered SGA, AGA, and LGA infants (FDP: 6.91±4.62 vs. 7.85±4.77 vs. 9.04±5.59 mg/L, HDL-C: 1.80±0.38 vs. 1.73±0.35 vs. 1.65±0.33 mmol/L, FHR: 4.02±3.20 vs. 4.70±3.30 vs. 5.72±3.76 mg/mmol; all $P < 0.01$; Fig. 2).

FHR and other parameters

Table 2 displays the correlation between FHR and other parameters. FHR was positively correlated with age ($r = 0.04$, $P < 0.01$), gestational age ($r = 0.07$, $P < 0.01$), triglyceride level ($r = 0.14$, $P < 0.01$), FDP level ($r = 0.93$, $P < 0.01$), and birthweight ($r = 0.20$, $P < 0.01$) and adversely correlated with systolic BP ($r = -0.05$, $P < 0.01$), diastolic BP (($r = -0.05$, $P < 0.01$), hsCRP level ($r = -0.07$, $P < 0.01$), total cholesterol level ($r = -0.06$, $P < 0.01$), and HDL-C level ($r = -0.4$, $P < 0.01$).

FHR and foetal growth indices and LGA/SGA

The associations of FDP, HDL-C, and FHR with foetal growth, and the delivery of LGA and SGA infants are presented in Table 3. Neonates born to women in Q4 of

FDP (> 9.60 mg/L) were 0.15 cm longer and 168 g heavier compared with those born to women in Q1 (< 4.67 mg/L). Pregnant women with HDL-C levels in the highest quartile (Q4: HDL-C > 1.94 mmol/L) had infants with a 69.47 g lower neonatal birth weight than pregnant women in the lowest quartile (Q1: HDL-C < 1.49 mmol/L). Compared with neonates born to women in the lowest quartile (Q1: FHR < 2.7) mg/mmol), neonates born to women with an FHR index above 5.90 mg/mmol (Q4) were 0.16 cm longer and 181 g heavier. The maternal FHR index was negatively associated with the delivery of SGA infants (OR, 0.76, 95% CI, 0.64, 0.91; OR, 0.48, 95% CI, 0.39, 0.58; and OR, 0.47, 95% CI, 0.38, 0.57; for Q 2, Q3, and Q4, respectively; *P* for trend < 0.01). The risk of delivering a LGA infant showed an increasing trend in association with women with an FHR in the second, third and fourth quartile (OR, 1.63, 95% CI, 1.37, 1.94; OR, 2.04, 95% CI, 1.71, 2.43; and OR, 2.85, 95% CI, 2.41, 3.38; respectively; *P* for trend < 0.01).

Combined impacts of maternal characteristics and FHR on LGA/ SGA

In subgroup analyses, the effects of other covariates on the association between FHR (≤5.9 vs. >5.9 mg/mmol) and the delivery of SGA/LGA infants were further evaluated. As presented in Table 4 and Table 5, the associations between elevated FHR and the delivery of SGA/LGA infants were consistent for the following subclassifications: age, BMI, parity, reproductive mode, and pregnancy complications, with the exception of PE on SGA (*P* for the interactions > 0.05). Notably, the incidence of SGA infants delivered by obese pregnant women (BMI ≥ 30 kg/m2) with high FHRs (>5.9 mg/mmol) was significantly lower than that of SGA infants

delivered by normal weight women (BMI < 25 kg/m2) with low FHRs (≤5.9 mg/mmol; 2.7% vs. 17.0%), indicating a 14.8% decrease in absolute risk and an 84% decrease in relative risk (OR, 0.16, 95% CI, 0.10, 0.28; *P* < 0.01). Additionally, the incidence of LGA infants delivered by obese pregnant women with high FHRs was significantly elevated compared with that of LGA infants delivered by obese pregnant women with normal weights and low FHRs (37.0% vs. 5.1%), indicating a 31.9% increase in absolute risk and an 8.8-fold relative risk (OR, 8.76, 95% CI, 6.68, 11.49; *P* < 0.01).

Predictive power of FHR and relevant models for SGA and LGA

To determine the cut-off points of FDP, HDL-C, FHR and related models for the prediction of delivering SGA and LGA infants, a ROC analysis was performed (Fig. 3). The AUCs of FHRs of women who delivered SGA and LGA infants were significantly higher than those of FDP and HDL-C (SGA: 0.587 vs. 0.577/0.544; LGA: 0.595 vs. 0.573/0.576 vs.; all *P* < 0.01, Tables 6). The optimal thresholds of FHR for predicting the delivery of SGA and LGA infants were 3.72 and 4.30 mg/mmol, with sensitivities of 59.96% and 56.92%, specificities of 53.96 and 56.69, negative predictive values of 92.00% and 86.53%, and positive predictive values of 13.25% and 21.21%, respectively. Additionally, two types of models incorporating clinical variables and laboratory indicators for predicting SGA and LGA were further investigated. Type 1 models included maternal age, BMI, BP, parity, gestational age, pregnancy complications, assisted reproduction, and neonatal sex and the levels of hsCRP and triglycerides, total cholesterol, and LDL-C. Type 2 models (FHR plus

Type 1) significantly increased the AUC of LGA from 0.725 to 0.739 ($P < 0.01$) and the AUC of SGA from 0.717 to 0.727 ($P < 0.01$).

Discussion

Main findings

In this pilot study, the maternal FHR index before labour was positively associated with LGA delivery and negatively associated with SGA delivery. The associations between FHR and LGA/SGA risk were markedly enhanced by BMI. In addition, FHR had a significantly higher AUC in predicting the delivery of LGA/SGA infants than FDP and HDL-C. Notably, these findings demonstrated that adding FHR into the traditional models could significantly increase the power of predicting the delivery of SGA and LGA infants.

Interpretation

Maternal TC, TG, and LDL-C levels increase gradually as the pregnancy progresses, reaching a peak in late pregnancy. In contrast, HDL-C concentration increases from early and middle pregnancy with a slight fall in late pregnancy [26,27]. Maternal physiological shifts from glucose metabolism to an increasing preference for lipid metabolism contributes to these changes [28]. The lipid metabolism is an important determinant of foetal growth. Until recently, it remains controversial that maternal disorders in lipid metabolism leads to foetal overgrowth in nondiabetic pregnancies. Several epidemiological studies revealed that there is a remarkable correlation between maternal dyslipidaemia during early-mid pregnancy with foetal birthweight and delivery of LGA infants [29–33]. However, results from a Turkish cohort study

indicated that none of the lipids (triglycerides/cholesterol/HDL-C/LDL-C) during the first trimester of pregnancy were related to the delivery of LGA infants [15]. Another cohort study from Canada further reported that among pregnant women without GDM, maternal leptin levels but not lipid levels (triglycerides/HDL-C/LDL-C), were related to the delivery of LGA infants during the late second or early third trimester of pregnancy [16]. In addition, the association during the third trimester of pregnancy appears to be more complex. Misra et al. in the US reported a significant negative association between HDL-C and neonatal birth weight during all trimesters of pregnancy only among overweight women (pregestational BMI ≥25 kg/m2), and found no significant associations between birth weight and lipids (triglycerides/cholesterol/HDL-C/LDL-C) in late gestation among normal weight women [34]. Kulkarni et al. in India further suggested that among thin women, HDL-C at both 18- and 28-week gestation were not associated with birth weight [35]. Mossayebi et al. in Iran argued that only triglyceride levels (not cholesterol/HDL-C/LDL-C levels) in late pregnancy (not early pregnancy), might be an independent predictor of LGA in women without GDM, obesity, and hypertension [36]. A previous cohort study revealed that both high triglycerides and low HDL-C (not total cholesterol/LDL-C) levels during late pregnancy are significantly and independently associated with high risk of delivering LGA infants among pregnant women with no complications, which are in line with previous studies from China [11–14,19,37,38]. From the authors' point of view, racial/ethnic differences might contribute to these conflicting results, which means that racial differences play an

important role in affecting a dynamic change of HDL-C levels during pregnancy and foetuses of different races might be disproportionately influenced by maternal lipid metabolism [39]. The current study further demonstrated that, among all Chinese women (those that are healthy and those that have pregnancy complications), HDL-C levels in the highest quartile (compared with the lowest quartile) in late pregnancy is significantly associated with deceased birthweight and with reduced risk of delivering LGA infants, but not with increased risk of delivering SGA infants (birthweight: adjusted $\beta = -69.47$ g, $P < 0.001$; LGA: adjusted $OR = 0.60$, $P < 0.001$; SGA: adjusted $OR = 1.15$, $P = 0.29$). Additionally, this study determined the predictive value of HDL-C to the delivery of LGA infants and SGA infants by using ROC analysis and found that the AUC of the optimal cut-point of < 1.73 mmol/L for the delivery of LGA infants is higher than that of the cut-point of > 1.89 mmol/L for the delivery of SGA infants (0.576 vs. 0.544).

It is generally believed that FDP level remains stable throughout pregnancy despite hypercoagulability during pregnancy [40]. However, a previous report demonstrated that FDP level progressively increases during late pregnancy until labour [19]. Increased FDP levels can not only be used to diagnose thrombosis and disseminated intravascular coagulation but also help to predict macrosomia/the delivery of LGA infants in normal pregnancies [17,19]. Thus, FDP investigated in this study to assess the birth weight as well as risk of delivering LGA/SGA infants, increasing the clinical significance of FDP measurement during late pregnancy. Unsurprisingly, the present study further revealed significant associations of elevated

FDP level with decreased risk of delivering SGA infants and increased risk of delivering LGA infants and for Q4 vs. Q1 among all Chinese women (LGA: adjusted OR = 2.50; SGA: adjusted OR = 0.46; all $P < 0.001$). In addition, ROC analysis suggests that FDP levels have a similar AUC for predicting the delivery of LGA or SGA infants (0.573 vs. 0.577). However, the AUC of the best cut-off values for SGA and LGA infants was relatively poor, suggesting that it is unsatisfactory for predicting these outcomes based on FDP or HDL-C levels alone. Then, FHR was introduced to improve the predictive power. Although FHR had a significantly greater AUC for predicting the delivery of SGA/LGA infants than FDP and HDL-C, the AUC was still unsatisfactory (for SGA: 0.587; for LGA: 0.595). The feasible predictive models based on prenatal clinical parameters may provide an opportunity to identify women at risk of delivering SGA/LGA infants. This study revealed that FHR, by itself, showed poor power for predicting the delivery of SGA/LGA infants in late pregnancies; however, adding FHR to the models based on prenatal parameters in late pregnancy increased the ability to predict these outcomes (for SGA: 0.727; for LGA: 0.739). In addition, until s the exact mechanism contributing to the association between the FHR and birth weight remains unclear.

Strengths and limitations

The strengths of this pilot study included the observational design, larger mother-and-singleton-offspring birth cohort, measurements of various parameters by trained professionals in the same specialized laboratory and sets of covariates. This study provides a better understanding of the combination of FDP and HDL-C (FHR),

which could improve the detection of foetal growth disorders and potentially provide an effective and inexpensive indicator to predict the risk of delivering SGA/LGA infants. To the best of the authors' knowledge, this is the first and largest study to determine associations between maternal FHR index and birthweight and delivery of LGA/SGA infants and to reveal the predictive value of FHR for the delivery of SGA/LGA infants in Chinese pregnant women. However, several limitations in this study should be mentioned. First, due to a lack of follow-up data on the clinical outcomes of offspring, whether FHR changes will affect long-term health outcomes and the occurrence of metabolic disease needs further study. Second, although multivariable adjusted regression models were applied, some uncollected or unknown factors might have an impact on SGA/LGA deliveries, such as maternal socioeconomic status, gestational weight gain, and history of GDM and SGA/LGA infant deliveries. Therefore, the effects of residual confounding factors cannot be excluded completely. Third, this is a retrospective single-centre pilot study, and selective bias is unavoidable. All of the subjects observed in this study were Chinese women, so the findings need to be confirmed in other ethnic populations.

Conclusions

In this study, the FHR was used as an innovative indicator of foetal birthweight to examine associations between the maternal FHR before labour and foetal growth outcomes. This study found that an elevated FHR is associated with increased birth length and weight of infants and risk of delivering LGA infants and associated with decreased risk of delivering SGA infants. Furthermore, the FHR combined with

traditional risk factors for delivering SGA and LGA infants have shown to increase predictive power. These findings suggested that the maternal FHR in late pregnancy could be a reliable and alternative indicator for predicting the delivery of LGA/SGA infants, representing an index worth taking into account when studying foetal birthweight-related changes. Considering the FHR in combination with routine risk factors is a simple and practical way to detect pregnant women at risk for delivering SGA/LGA infants, which may be helpful in a clinical setting and in obstetric management.

Manuscript for iThenticate.docx

ORIGINALITY REPORT

15%
SIMILARITY INDEX

PRIMARY SOURCES

1	www.science.gov<br>Internet	65 words — 2%
2	www.ncbi.nlm.nih.gov<br>Internet	63 words — 1%
3	bmcpublichealth.biomedcentral.com<br>Internet	58 words — 1%
4	Ather Mansoor. "Echocardiographic Determination of Epicardial Adipose Tissue in Healthy Bonnet Macaques", Echocardiography, 02/2010<br>Crossref	54 words — 1%
5	Kana Serizawa, Kohei Ogawa, Naoko Arata, Atsushi Ogihara, Reiko Horikawa, Naoko Sakamoto. "Association between low maternal low-density lipoprotein cholesterol levels in the second trimester and delivery of small for gestational age infants at term: a case-control study of the national center for child health and development birth cohort", The Journal of Maternal-Fetal & Neonatal Medicine, 2016<br>Crossref	41 words — 1%
6	Xiaosong Yuan, Jing Wang, Yanfang Gao, Huiyan Wang, Bin Yu. "Impact of maternal thyroid hormone in late pregnancy on adverse birth outcomes: A retrospective cohort study in China", Endocrine Journal, 2020<br>Crossref	25 words — 1%

7	"American Transplant Congress 2007 Executive and Program Planning Committees and Abstract Review Committees", American Journal of Transplantation, 5/2007<br>Crossref	24 words — 1%
8	Xiaosong Yuan, Yanfang Gao, Ming Zhang, Wei Long, Jianbing Liu, Huiyan Wang, Bin Yu, Jun Xu. "Fibrin/fibrinogen degradation products in late pregnancy promote macrosomia prediction in normal uncomplicated pregnancy", Placenta, 2020<br>Crossref	24 words — 1%
9	www.omicsdi.org<br>Internet	22 words — 1%
10	"Abtracts from the 30th Annual Meeting of the Society of General Internal Medicine", Journal of General Internal Medicine, 2007<br>Crossref	20 words — < 1%
11	Xiaosong Yuan, Lingna Zhou, Bin Zhang, Huiyan Wang, Bin Yu, Jun Xu. "Association between low fetal fraction of cell free DNA at the early second-trimester and adverse pregnancy outcomes", Pregnancy Hypertension, 2020<br>Crossref	17 words — < 1%
12	Yaqoot Fatima, Suhail A.R. Doi, Abdullah Al Mamun. "Sleep problems in adolescence and overweight/obesity in young adults: is there a causal link?", Sleep Health, 2018<br>Crossref	17 words — < 1%
13	worldwidescience.org<br>Internet	17 words — < 1%
14	Panpan Ye, Wen Xu, Xiajing Tang, Ke Yao, Zhaochun Li, Hesheng Xu, Junting Shi.	16 words — < 1%

"Conductive keratoplasty for symptomatic presbyopia following monofocal intraocular lens implantation", Clinical & Experimental Ophthalmology, 2011
Crossref

15 Hugh M. Ehrenberg, Brian M. Mercer, Patrick M. Catalano. "The influence of obesity and diabetes on the prevalence of macrosomia", American Journal of Obstetrics and Gynecology, 2004 — 14 words — < 1%
Crossref

16 Vaibhav Patil, Raghvendra Bohara, Carla Winter, Michelle Kilcoyne, Siobhan McMahon, Abhay Pandit. "An insight into new glycotherapeutic in glial inflammation: Understanding the role of glycosylation from acute to chronic phase of inflammation", Cold Spring Harbor Laboratory, 2021 — 13 words — < 1%
Crossref Posted Content

17 academic.oup.com — 12 words — < 1%
Internet

18 journals.lww.com — 12 words — < 1%
Internet

19 Jain, Arun P., Jeffrey A. Gavard, Jim J. Rice, Rosemary B. Catanzaro, Raul Artal, and Sarah A. Hopkins. "The Impact of Interpregnancy Weight Change on Birthweight in Obese Women :", Obstetrical & Gynecological Survey, 2013. — 11 words — < 1%
Crossref

20 Reiss, K., J. Breckenkamp, T. Borde, S. Brenne, M. David, and O. Razum. "Contribution of overweight and obesity to adverse pregnancy outcomes among immigrant and non-immigrant women in Berlin, Germany", The European Journal of Public Health, 2015. — 11 words — < 1%

Crossref

21	topsecretapiaccess.dovepress.com<br>Internet	11 words — < 1%
22	www.researchgate.net<br>Internet	11 words — < 1%
23	AAAMJ Djelantik. "Contribution of overweight and obesity to the occurrence of adverse pregnancy outcomes in a multi-ethnic cohort: population attributive fractions for Amsterdam : Overweight and the PAF of adverse pregnancy outcomes", BJOG An International Journal of Obstetrics & Gynaecology, 02/2012<br>Crossref	10 words — < 1%
24	Djelantik, A.A., A.E. Kunst, M.F. van der Wal, H.A. Smit, and T.G. Vrijkotte. "Contribution of Overweight and Obesity to the Occurrence of Adverse Pregnancy Outcomes in a Multiethnic Cohort : Population Attributive Fractions for Amsterdam", Obstetric Anesthesia Digest, 2013.<br>Crossref	10 words — < 1%
25	journals.plos.org<br>Internet	10 words — < 1%
26	Halina I. Falfushynska, Lesya L. Gnatyshyna, Anna V. Ivanina, Inna M. Sokolova, Oksana B. Stoliar. "Detoxification and cellular stress responses of unionid mussels Unio tumidus from two cooling ponds to combined nano-ZnO and temperature stress", Chemosphere, 2018<br>Crossref	9 words — < 1%
27	Guillermo Gosebruch, Mauricio Sánchez, Jorge Delva, Fernando Wagner, James C. Anthony. "Family Attention and Tobacco Smoking Among Adolescents in	8 words — < 1%

Central America, Panama, and the Dominican Republic", Substance Use & Misuse, 2009
Crossref

28 Livia Costa de Oliveira, Ana Beatriz Franco-Sena, Fernanda Rebelo, Dayana Rodrigues Farias et al. "Factors associated with maternal serum C-reactive protein throughout pregnancy: A longitudinal study in women of Rio de Janeiro, Brazil", Nutrition, 2015 — 8 words — < 1%
Crossref

29 Makoto Saito, Kazunori Nishimura, Hiroki Nozue, Yayoi Miyazono, Tomohiro Kamoda. "Changes in Serum Adiponectin Levels from Birth to Term-Equivalent Age Are Associated with Postnatal Weight Gain in Preterm Infants", Neonatology, 2011 — 8 words — < 1%
Crossref

30 ecmj.org.cn — 8 words — < 1%
Internet

31 www.medrxiv.org — 8 words — < 1%
Internet

EXCLUDE QUOTES	OFF	EXCLUDE SOURCES	OFF
EXCLUDE BIBLIOGRAPHY	ON	EXCLUDE MATCHES	OFF
